# Walking

**Published:** 1892-11

**Authors:** 


### Walking.

Walking, if properly and regularly followed, would become not only a restorer of health to many who to-day are on the road to disease, but also a source of pleasure. Let the arms swing if you feel like it, and the limbs, too; open the nostrils and fill the lungs, and the movements will send a gentle electric vibration through the entire body, the result of which is the awakening of new life.

Never take the lazy gait, as it soon makes one tired, and produces languor. A little perspiration on the "home stretch" may prove to be a blessing, not only in carrying effete matter from the body, but in bringing an increased supply of oxygen into the blood, and putting the blush of health upon the cheek.

Perhaps the best time to walk is in the early morning. The air is then the most highly charged with the life-giving oxygen, and the freest from dust, smoke, etc., of traffic, which rises later in the day. At this time also the mind is liable to be more free from worry and anxiety, hence in the best condition to drink in the blessings of freshness for us on every hand.

